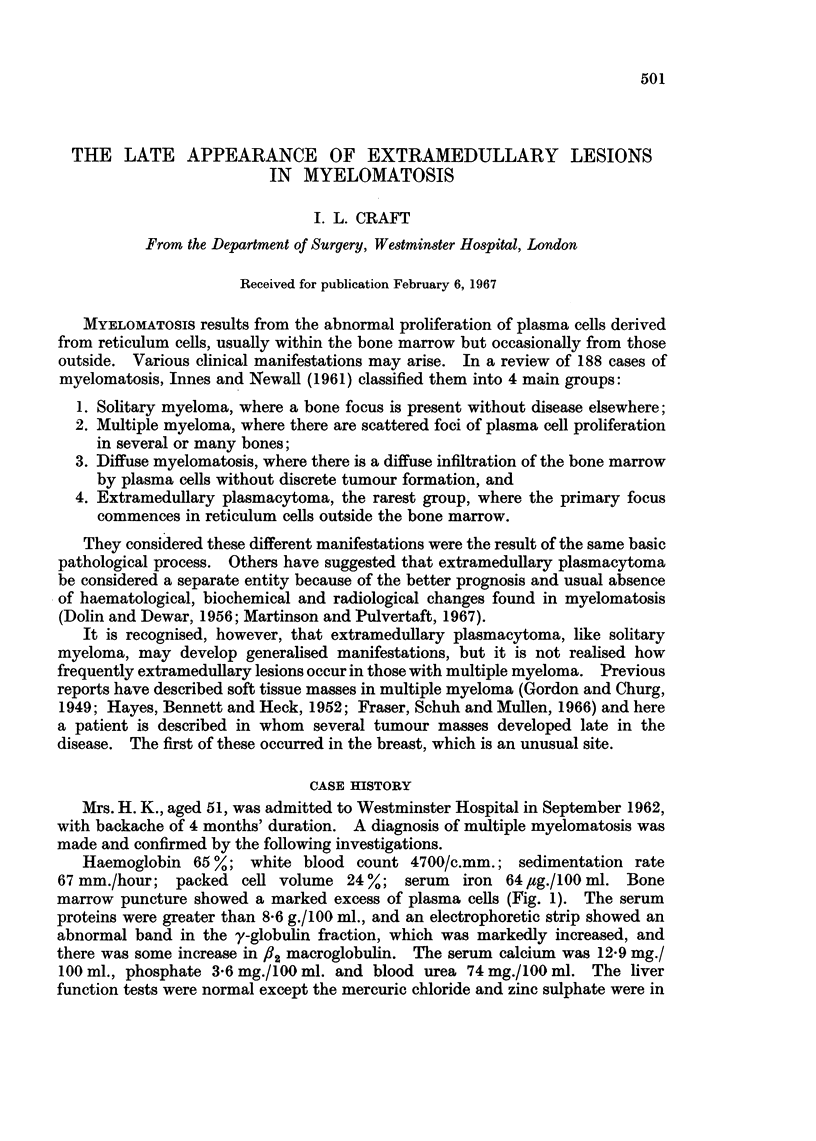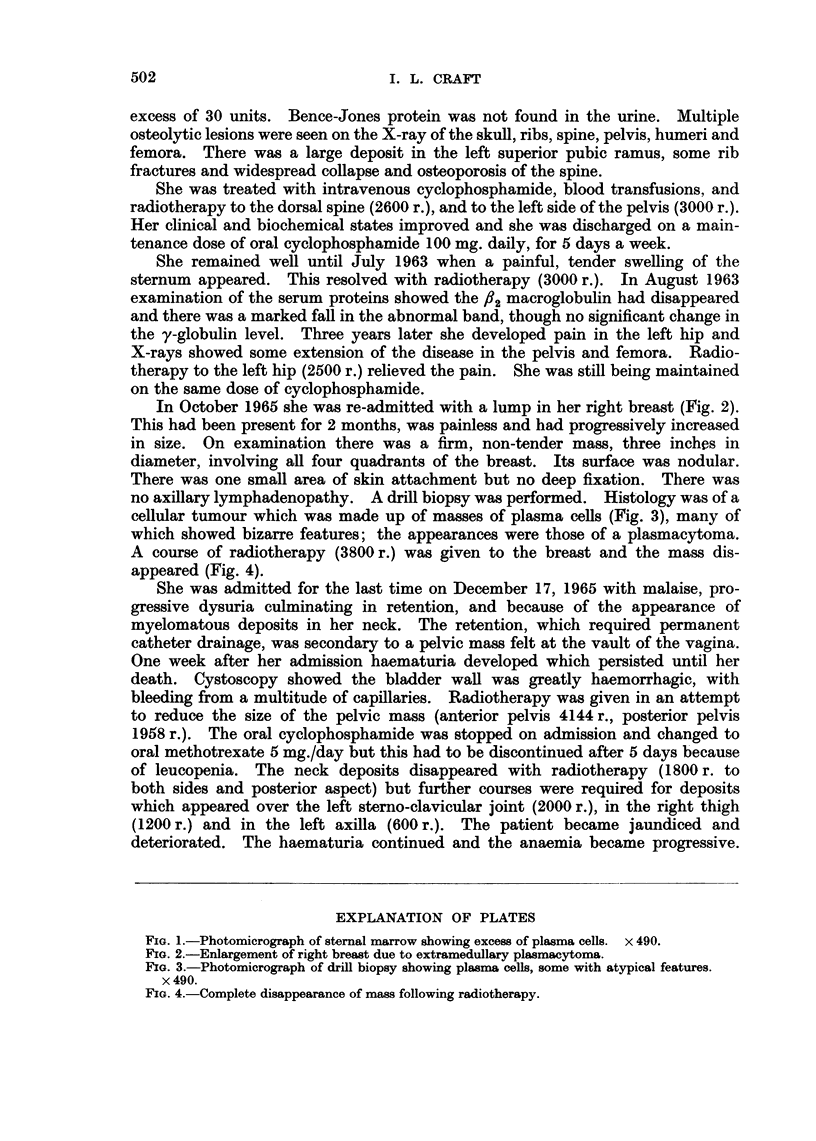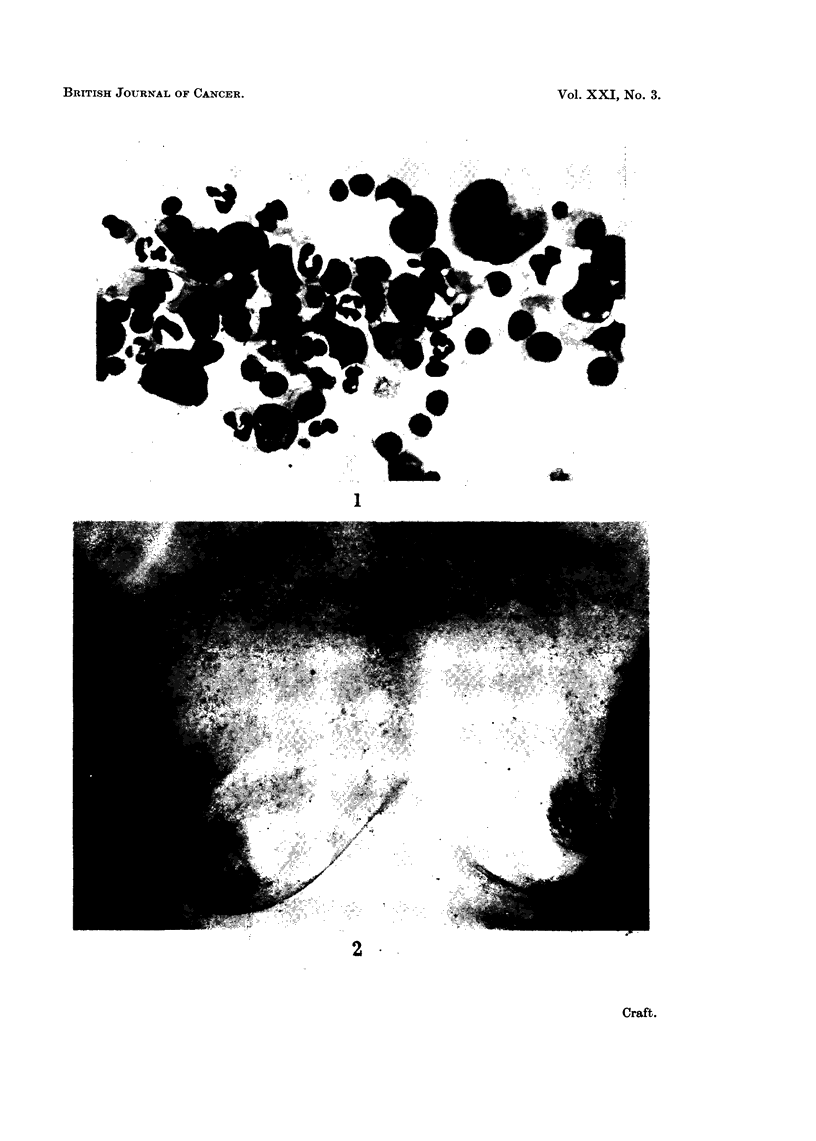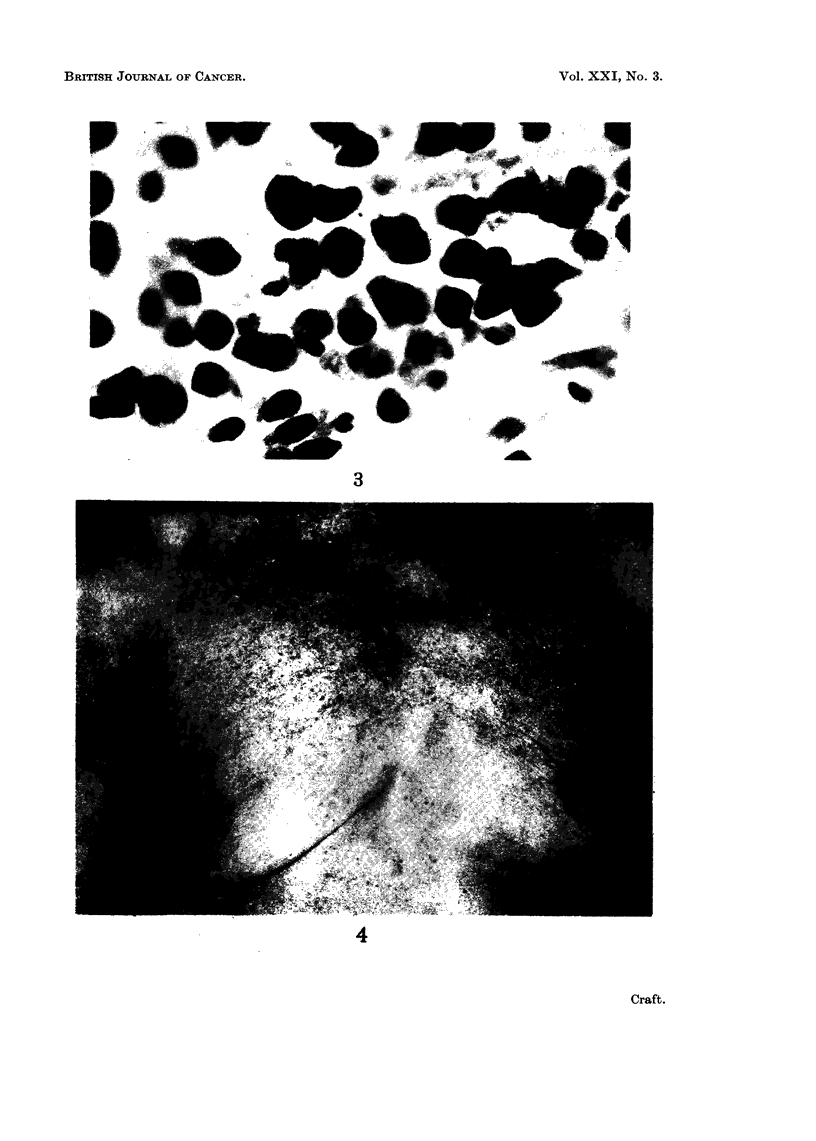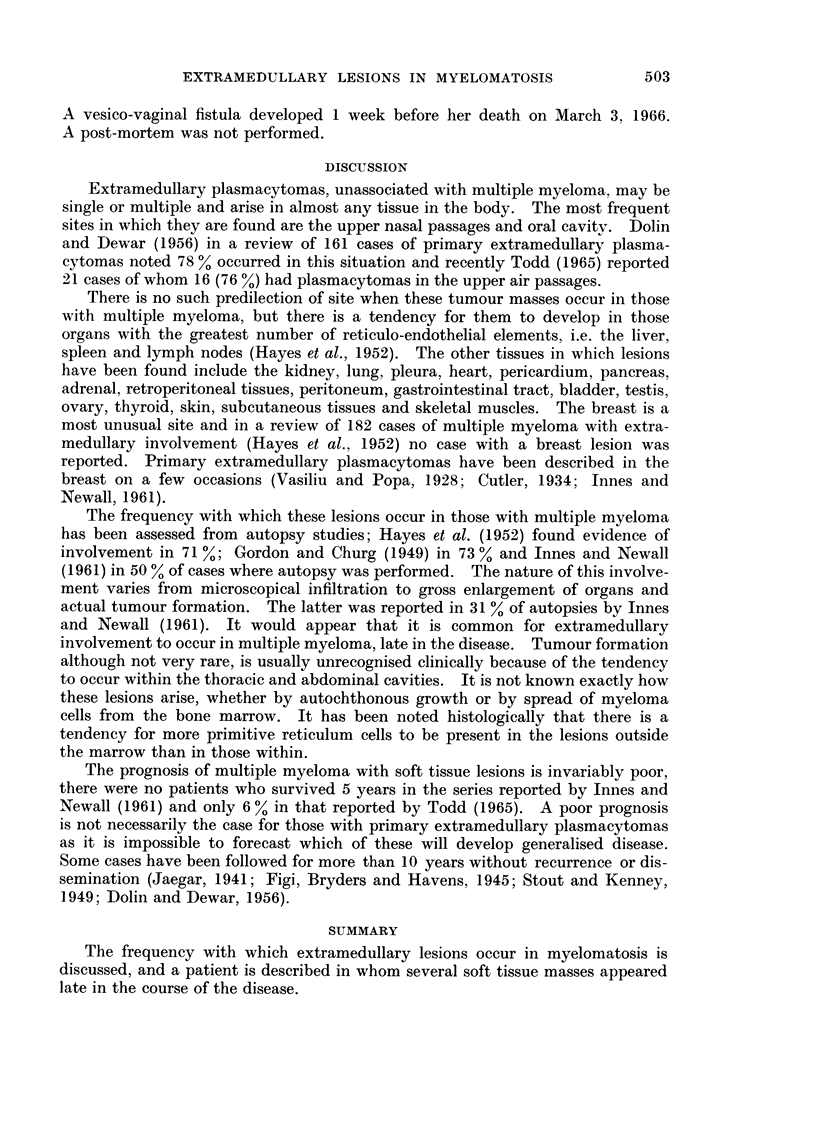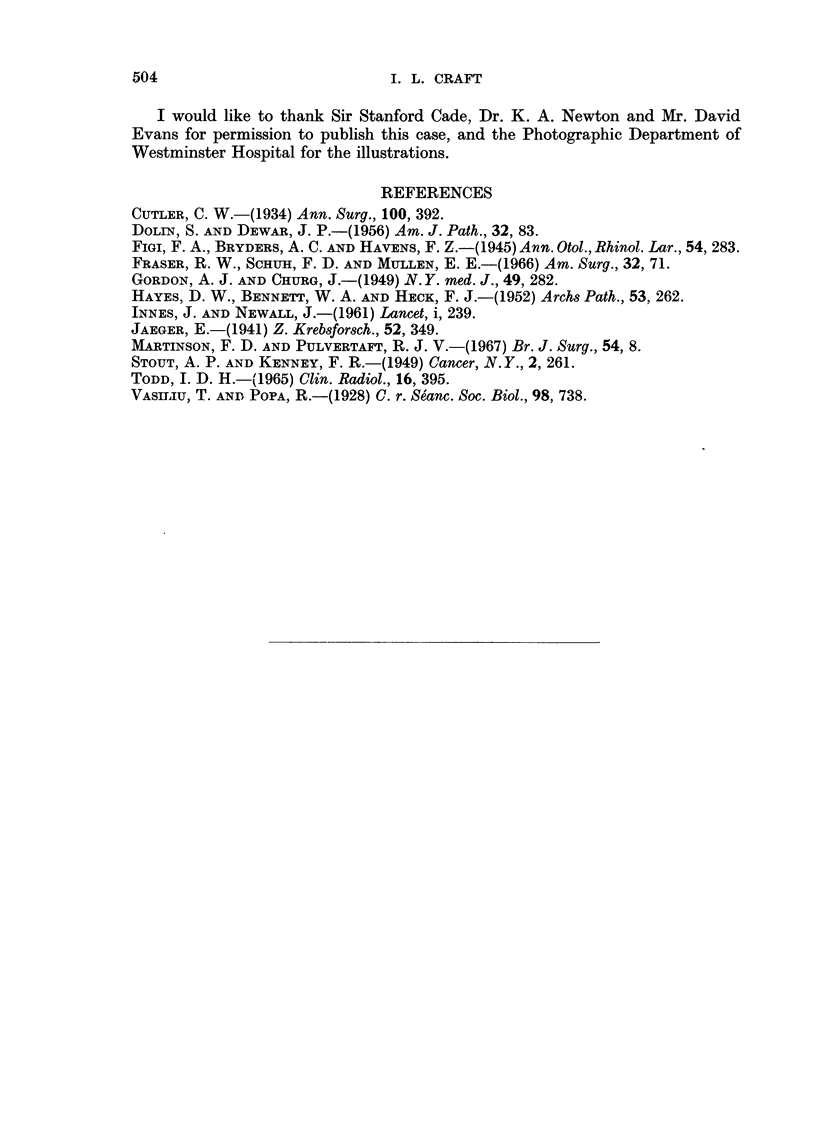# The late appearance of extramedullary lesions in myelomatosis.

**DOI:** 10.1038/bjc.1967.58

**Published:** 1967-09

**Authors:** I. L. Craft

## Abstract

**Images:**


### THE LATE APPEARANCE OF EXTRAMEDULLARY LESIONS IN MYELOMATOSIS

I. L. CRAFT

*From the Department of Surgery, Westminster Hospital, London*

Received for publication February 6, 1967

MYELOMATOSIS results from the abnormal proliferation of plasma cells derived from reticulum cells, usually within the bone marrow but occasionally from those outside. Various clinical manifestations may arise. In a review of 188 cases of myelomatosis, Innes and Newall (1961) classified them into 4 main groups:

1. Solitary myeloma, where a bone focus is present without disease elsewhere;
2. Multiple myeloma, where there are scattered foci of plasma cell proliferation in several or many bones;
3. Diffuse myelomatosis, where there is a diffuse infiltration of the bone marrow by plasma cells without discrete tumour formation, and
4. Extramedullary plasmacytoma, the rarest group, where the primary focus commences in reticulum cells outside the bone marrow.

They considered these different manifestations were the result of the same basic pathological process. Others have suggested that extramedullary plasmacytoma be considered a separate entity because of the better prognosis and usual absence of haematological, biochemical and radiological changes found in myelomatosis (Dolin and Dewar, 1956; Martinson and Pulvertaft, 1967).

It is recognised, however, that extramedullary plasmacytoma, like solitary myeloma, may develop generalised manifestations, but it is not realised how frequently extramedullary lesions occur in those with multiple myeloma. Previous reports have described soft tissue masses in multiple myeloma (Gordon and Churg, 1949; Hayes, Bennett and Heck, 1952; Fraser, Schuh and Mullen, 1966) and here a patient is described in whom several tumour masses developed late in the disease. The first of these occurred in the breast, which is an unusual site.

### CASE HISTORY

Mrs. H. K., aged 51, was admitted to Westminster Hospital in September 1962, with backache of 4 months' duration. A diagnosis of multiple myelomatosis was made and confirmed by the following investigations.

Haemoglobin 65%; white blood count 4700/c.mm.; sedimentation rate 67 mm./hour; packed cell volume 24%; serum iron 64 $\mu$g./100 ml. Bone marrow puncture showed a marked excess of plasma cells (Fig. 1). The serum proteins were greater than 8·6 g./100 ml., and an electrophoretic strip showed an abnormal band in the $\gamma$-globulin fraction, which was markedly increased, and there was some increase in $\beta_2$ macroglobulin. The serum calcium was 12·9 mg./100 ml., phosphate 3·6 mg./100 ml. and blood urea 74 mg./100 ml. The liver function tests were normal except the mercuric chloride and zinc sulphate were in excess of 30 units. Bence-Jones protein was not found in the urine. Multiple osteolytic lesions were seen on the X-ray of the skull, ribs, spine, pelvis, humeri and femora. There was a large deposit in the left superior pubic ramus, some rib fractures and widespread collapse and osteoporosis of the spine.

She was treated with intravenous cyclophosphamide, blood transfusions, and radiotherapy to the dorsal spine (2600 r.), and to the left side of the pelvis (3000 r.). Her clinical and biochemical states improved and she was discharged on a maintenance dose of oral cyclophosphamide 100 mg. daily, for 5 days a week.

She remained well until July 1963 when a painful, tender swelling of the sternum appeared. This resolved with radiotherapy (3000 r.). In August 1963 examination of the serum proteins showed the $\beta_2$ macroglobulin had disappeared and there was a marked fall in the abnormal band, though no significant change in the $\gamma$-globulin level. Three years later she developed pain in the left hip and X-rays showed some extension of the disease in the pelvis and femora. Radiotherapy to the left hip (2500 r.) relieved the pain. She was still being maintained on the same dose of cyclophosphamide.

In October 1965 she was re-admitted with a lump in her right breast (Fig. 2). This had been present for 2 months, was painless and had progressively increased in size. On examination there was a firm, non-tender mass, three inches in diameter, involving all four quadrants of the breast. Its surface was nodular. There was one small area of skin attachment but no deep fixation. There was no axillary lymphadenopathy. A drill biopsy was performed. Histology was of a cellular tumour which was made up of masses of plasma cells (Fig. 3), many of which showed bizarre features; the appearances were those of a plasmacytoma. A course of radiotherapy (3800 r.) was given to the breast and the mass disappeared (Fig. 4).

She was admitted for the last time on December 17, 1965 with malaise, progressive dysuria culminating in retention, and because of the appearance of myelomatous deposits in her neck. The retention, which required permanent catheter drainage, was secondary to a pelvic mass felt at the vault of the vagina. One week after her admission haematuria developed which persisted until her death. Cystoscopy showed the bladder wall was greatly haemorrhagic, with bleeding from a multitude of capillaries. Radiotherapy was given in an attempt to reduce the size of the pelvic mass (anterior pelvis 4144 r., posterior pelvis 1958 r.). The oral cyclophosphamide was stopped on admission and changed to oral methotrexate 5 mg./day but this had to be discontinued after 5 days because of leucopenia. The neck deposits disappeared with radiotherapy (1800 r. to both sides and posterior aspect) but further courses were required for deposits which appeared over the left sterno-clavicular joint (2000 r.), in the right thigh (1200 r.) and in the left axilla (600 r.). The patient became jaundiced and deteriorated. The haematuria continued and the anaemia became progressive.

---

EXPLANATION OF PLATES

FIG. 1.—Photomicrograph of sternal marrow showing excess of plasma cells. ×490.

FIG. 2.—Enlargement of right breast due to extramedullary plasmacytoma.

FIG. 3.—Photomicrograph of drill biopsy showing plasma cells, some with atypical features. ×490.

FIG. 4.—Complete disappearance of mass following radiotherapy.

1

2

Craft.

3

4

Craft.

A vesico-vaginal fistula developed 1 week before her death on March 3, 1966. A post-mortem was not performed.

### DISCUSSION

Extramedullary plasmacytomas, unassociated with multiple myeloma, may be single or multiple and arise in almost any tissue in the body. The most frequent sites in which they are found are the upper nasal passages and oral cavity. Dolin and Dewar (1956) in a review of 161 cases of primary extramedullary plasmacytomas noted 78 % occurred in this situation and recently Todd (1965) reported 21 cases of whom 16 (76 %) had plasmacytomas in the upper air passages.

There is no such predilection of site when these tumour masses occur in those with multiple myeloma, but there is a tendency for them to develop in those organs with the greatest number of reticulo-endothelial elements, i.e. the liver, spleen and lymph nodes (Hayes *et al.*, 1952). The other tissues in which lesions have been found include the kidney, lung, pleura, heart, pericardium, pancreas, adrenal, retroperitoneal tissues, peritoneum, gastrointestinal tract, bladder, testis, ovary, thyroid, skin, subcutaneous tissues and skeletal muscles. The breast is a most unusual site and in a review of 182 cases of multiple myeloma with extramedullary involvement (Hayes *et al.*, 1952) no case with a breast lesion was reported. Primary extramedullary plasmacytomas have been described in the breast on a few occasions (Vasiliu and Popa, 1928; Cutler, 1934; Innes and Newall, 1961).

The frequency with which these lesions occur in those with multiple myeloma has been assessed from autopsy studies; Hayes *et al.* (1952) found evidence of involvement in 71 %; Gordon and Churg (1949) in 73 % and Innes and Newall (1961) in 50 % of cases where autopsy was performed. The nature of this involvement varies from microscopical infiltration to gross enlargement of organs and actual tumour formation. The latter was reported in 31 % of autopsies by Innes and Newall (1961). It would appear that it is common for extramedullary involvement to occur in multiple myeloma, late in the disease. Tumour formation although not very rare, is usually unrecognised clinically because of the tendency to occur within the thoracic and abdominal cavities. It is not known exactly how these lesions arise, whether by autochthonous growth or by spread of myeloma cells from the bone marrow. It has been noted histologically that there is a tendency for more primitive reticulum cells to be present in the lesions outside the marrow than in those within.

The prognosis of multiple myeloma with soft tissue lesions is invariably poor, there were no patients who survived 5 years in the series reported by Innes and Newall (1961) and only 6 % in that reported by Todd (1965). A poor prognosis is not necessarily the case for those with primary extramedullary plasmacytomas as it is impossible to forecast which of these will develop generalised disease. Some cases have been followed for more than 10 years without recurrence or dissemination (Jaegar, 1941; Figi, Bryders and Havens, 1945; Stout and Kenney, 1949; Dolin and Dewar, 1956).

### SUMMARY

The frequency with which extramedullary lesions occur in myelomatosis is discussed, and a patient is described in whom several soft tissue masses appeared late in the course of the disease.

I would like to thank Sir Stanford Cade, Dr. K. A. Newton and Mr. David Evans for permission to publish this case, and the Photographic Department of Westminster Hospital for the illustrations.

### REFERENCES

CUTLER, C. W.—(1934) *Ann. Surg.*, **100**, 392.
DOLIN, S. AND DEWAR, J. P.—(1956) *Am. J. Path.*, **32**, 83.
FIGI, F. A., BRYDERS, A. C. AND HAVENS, F. Z.—(1945) *Ann. Otol., Rhinol. Lar.*, **54**, 283.
FRASER, R. W., SCHUH, F. D. AND MULLEN, E. E.—(1966) *Am. Surg.*, **32**, 71.
GORDON, A. J. AND CHURG, J.—(1949) *N.Y. med. J.*, **49**, 282.
HAYES, D. W., BENNETT, W. A. AND HECK, F. J.—(1952) *Archs Path.*, **53**, 262.
INNES, J. AND NEWALL, J.—(1961) *Lancet*, i, 239.
JAEGER, E.—(1941) *Z. Krebsforsch.*, **52**, 349.
MARTINSON, F. D. AND PULVERTAFT, R. J. V.—(1967) *Br. J. Surg.*, **54**, 8.
STOUT, A. P. AND KENNEY, F. R.—(1949) *Cancer, N.Y.*, **2**, 261.
TODD, I. D. H.—(1965) *Clin. Radiol.*, **16**, 395.
VASILIU, T. AND POPA, R.—(1928) *C. r. Séanc. Soc. Biol.*, **98**, 738.